# Effects of Housing Aid on Psychosocial Health after a Disaster

**DOI:** 10.3390/ijerph19127302

**Published:** 2022-06-14

**Authors:** Maria M. Laurito, Elizabeth Frankenberg, Duncan Thomas

**Affiliations:** 1 Analysis Group, Inc., Boston, MA 02199, USA; maria.laurito@duke.edu; 2 Department of Sociology and the Carolina Population Center, University of North Carolina at Chapel Hill, Chapel Hill, NC 27516, USA; 3 Department of Economics, Duke University, Durham, NC 27708, USA; dthomas@econ.duke.edu

**Keywords:** natural disaster, reconstruction, housing aid, psychological well-being, Indonesia

## Abstract

Little is known about whether the provision of aid in the aftermath of a large-scale natural disaster affects psychological well-being. We investigate the effects of housing assistance, a key element of the reconstruction program implemented after the 2004 Indian Ocean tsunami. Population-representative individual-level longitudinal data collected in Aceh, Indonesia, during the decade after the tsunami as part of the Study of the Tsunami Aftermath and Recovery (STAR) are used. Housing aid was targeted to people whose homes were destroyed and, to a lesser extent, damaged by the tsunami and to those who lived, at the time of the tsunami, in communities that sustained the greatest damage. The effects of receipt of aid on post-traumatic stress reactivity (PTSR) are examined using panel data models that take into account observed and unobserved individual-specific fixed characteristics that affect both PTSR and aid receipt, drawing comparisons in each survey wave between individuals who had been living in the same *kecamatan* when the tsunami hit. Those who received aid have better psychological health; the effects increase with time since aid receipt and are the greatest at two years or longer after the receipt. The effects are concentrated among those whose homes were destroyed in the tsunami.

## 1. Introduction

Natural disasters are increasing in intensity and frequency across the globe, causing extensive damage to the built and natural environment and imposing enormous hardships on affected populations. Disasters kill people, sweep away communities, destroy livelihoods and assets, and upend economic opportunities [1,2]. The stressors caused by natural disasters have been implicated in a wide array of poor social, economic, health, and demographic outcomes for individuals and their families not only in the immediate aftermath of the disaster but, in some cases, over the longer-term as well [3,4,5,6,7,8,9,10]. Evidence is limited regarding the extent to which these impacts are mitigated by recovery programs. Our paper contributes to filling this important gap in the science.

Focusing on psychological health both immediately after a large-scale natural disaster and over the following ten years, our research examines links between the evolution of this dimension of population health and the provision of housing assistance. The context for our work is the Indonesian province of Aceh after the 2004 Indian Ocean tsunami. We used data from the Study of the Tsunami Aftermath and Recovery (STAR). Housing aid, a key element of the post-tsunami recovery and reconstruction program, was targeted to those whose homes were damaged or destroyed.

Understanding the impacts of housing reconstruction is important both because housing programs absorb substantial shares of post-disaster assistance funding and because one of the ways through which disasters affect psychosocial health is through damage and destruction of housing and the disruption to individual and community life that ensues [11,12,13,14,15]. Research in a variety of settings has documented that housing disruption after a disaster is associated with greater psychological distress [16,17,18,19]. 

Many potential pathways underly the association between housing damage and psychosocial health, and the precise mechanism is not clear. First, housing is a major economic asset, accounting for a large share of family wealth in many cases. Second, housing and residential location may be important sources of identity and connection with community [20]. Third, housing disruptions are directly stressful and also may be accompanied by disruption of family and social networks and stability and by loss of livelihoods and of economic and educational opportunities [21]. Loss of assets, disruption of networks, and the stress of identifying a safe place to live may all drive poor psychological health.

Provision of emergency shelter is often part of humanitarian programs introduced after a disaster, but re-establishing populations in more permanent housing falls between relief and development, with fewer guidelines and best practices associated with its provision than with emergency humanitarian assistance [20,22]. Research has documented links between housing conditions after a disaster and psychological distress across a range of contexts. Several studies have focused on Japan in the aftermath of the Great East Japan Earthquake of 2011, all finding that temporary or uncertain housing is associated with higher risks of psychological distress [18,23,24]. Similar results characterize individuals affected by cyclone Amphan in Bangladesh [25] and individuals who experienced lengthy patterns of unstable housing after Hurricane Katrina in the United States [17]. The findings point to the important role that housing plays as an asset and as a source of connection to community and to opportunities of various forms and the stress created by housing uncertainty.

In contrast with this literature, relatively little is known about whether the provision of housing assistance affects health outcomes and if so, over what time frame. We provide empirical evidence on both these questions, which are important given the increasing frequency and intensity of highly destructive disasters in combination with the costs associated with provision of housing. 

Our work is set in Indonesia. On 26 December 2004, a powerful earthquake off the coast of the island of Sumatra generated a series of tsunami waves that ultimately reached the shorelines of 26 countries bordering the Indian Ocean. The province of Aceh was hit hardest. The death toll is estimated at 167,000 people [26], which was about five percent of the population of the province at the time. Over 200,000 houses were damaged or destroyed, and over 700,000 people were displaced [27,28]. Initial tallies of economic losses exceeded USD 4.5 billion—almost the GDP of the entire province in the year before the tsunami [29]. 

The tsunami resulted in an unprecedented outpouring of financial support from governments, aid agencies, international and domestic NGOs, and private citizens. Pledges to Indonesia totaled more than USD 7 billion [30]. Of the total amount committed to Indonesia, USD 1.5 billion were in excess of the estimated cost of the reconstruction, which allowed the Indonesian government to set the goal of “building back better”. The needs of the housing sector were enormous, with about USD 1.6 billion of the pledges set aside for housing reconstruction and repair [31]. Under the coordination of the Indonesian government, the reconstruction brought together multiple players, including multilateral organizations, foreign donors, and domestic and international NGOs [31].

Before the tsunami, Aceh was the site of a long-running conflict between the Free Aceh Movement (GAM) and the Indonesian military, which was particularly disruptive in the years just before the tsunami [26]. The security situation remained tense in the immediate aftermath of the tsunami, which complicated efforts to provide humanitarian assistance. However, in August 2005, GAM and the Government of Indonesia signed a peace agreement, known as the Helsinki Accords, greatly facilitating the recovery effort and reducing tensions throughout the province [32]. 

The reconstruction process was carried out in roughly three phases. First, assistance programs concentrated on providing immediate relief for basic needs, such as food, shelter, and access to clean water [26]. In April 2005, the second phase began, which emphasized reconstruction and development, including housing, health, transportation, water and sanitation, and programs designed to promote rehabilitation of livelihoods [33]. Starting some two years later, the third phase began with the goal of rebuilding the economy and the government [26].

To coordinate the reconstruction effort, the government of Indonesia created the Agency for Reconstruction and Rehabilitation (BRR), making it responsible directly to the President, which simplified regulations and procurement processes [34]. BRR attracted highly qualified professionals who developed financial management and monitoring tools to track the progress of the many projects underway simultaneously [35]. Ultimately, BRR oversaw more than 2000 projects distributed by over 400 organizations [31]. Around 120 NGOs participated in housing reconstruction, which entailed 266 separate projects [36]. 

Central to the Government of Indonesia’s housing reconstruction and rehabilitation policy was the provision of assistance to those whose homes had been affected by the disaster. Assistance took the form of a standard 36 m^2^ home for those owners whose homes were completely destroyed and, for those who homes were damaged, funds for rehabilitation. The policy directive also provided assistance to renters rendered homeless by the tsunami, which was equivalent to about 40 percent of the value of a 36 m^2^ home [37]. The prototype structure, intended to be suitable for a family of four, consisted of a living room, a kitchen, two bedrooms, and a toilet. The policy was structured so that houses were rebuilt in the same location as the pre-tsunami house wherever possible to avoid disputes over land property rights. 

Several factors complicated the process of providing housing. Land titles were one complication. Because the tsunami changed much of the physical landscape and destroyed legal documentation of ownership, in August 2005, the Reconstruction of Aceh Land Administration System (RALAS) was launched [32]. By December 2006, RALAS had measured 134,300 land parcels and signed 17,400 land titles [38]. Another complication arose around accessing timber, which involved trade-offs between prices and the possibility of inadvertently encouraging illegal logging [32].

**Figure 1:**
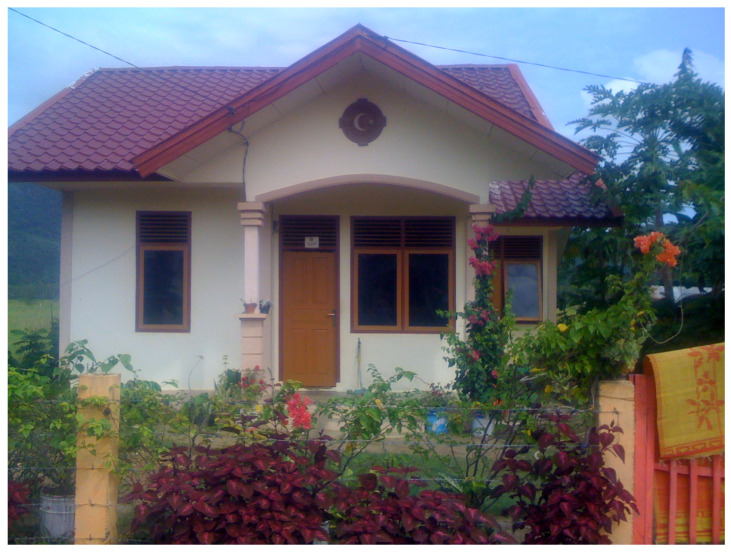
Tsunami assistance house (author’s photo).

Housing construction began slowly, in part because of the need to address land ownership and in part because of a desire to engage communities in the planning process and to emphasize quality and sustainability of construction [26]. These goals reflected the “build back better” mantra that guided BRR and NGO efforts, which in Aceh did not involve large-scale resettlement away from the ocean. Construction accelerated during late 2005 and 2006. As of September 2005, about 7000 families had received new homes, while an estimated 59,000 families had returned to their old properties even though houses had not yet been built [29]. The pace of housing construction began to ramp up, with 16,000 houses complete by December 2005. In 2006, some 40,000 more were constructed, but BRR’s ambitious target of 90,000–100,000 by the end of that year was not achieved [32]. As of December 2007, NGOs had disbursed some 56% of their housing funds (across 219 projects) in comparison with about 80% of government and donor funds (across 47 projects), resulting in 114,000 houses constructed by July 2008 [31]. A prototypical house is displayed in Figure 1.

In the first three years after the tsunami, survivors worked to put their lives back together. When the waves came ashore, many experienced the terror of trying to escape the waves or witnessed friends and family members drown. Damage was widespread, and many individuals lived for months or years amongst reminders impossible to avoid in communities that had been reduced to rubble. It is plausible in this context that receipt of an assistance home substantially reduced the difficulties that the affected populations faced. To that end, we analyzed whether receipt of housing assistance affects psychosocial health among disaster survivors.

## 2. Materials and Methods

### 2.1. Data

Data were drawn from the Study of the Tsunami Aftermath and Recovery (STAR), an on-going longitudinal survey of individuals, their households, and their communities in two provinces, Aceh and North Sumatra, on the island of Sumatra, Indonesia [39]. Data that serve as the pre-tsunami baseline for STAR was provided by Statistics Indonesia, who routinely collect a large-scale *Survei Sosial Ekonomi Nasional* (National Socio-economic Survey, SUSENAS). Designed to provide information on the evolution of household well-being in the country, at the time of the tsunami, SUSENAS was collected annually, and in each round, a new cross-section of households was drawn that was representative of every *kabupaten* (district) in Indonesia. The STAR baseline comprises respondents included in the February/March 2004 round of SUSENAS who were interviewed 10 months before the tsunami. The baseline includes 26,900 individuals who were living in 405 enumeration areas along the coast of Aceh and the west coast of North Sumatra. To assure the STAR baseline is population representative, all enumeration areas in *kabupaten* that had a coastline that was vulnerable to the tsunami waves were included in the sample. 

Fieldwork for the first STAR follow-up survey started five months after the tsunami in May 2005 and lasted for 13 months. We stayed in the field continuously for the next four years and conducted four more annual follow-ups, completing the fifth follow-up in May 2010. Our ten-year follow-up took place in 2014–2015. Data from these six follow-ups were used in this research. A fifteen-year follow-up is in progress, having been delayed by COVID.

Every member of the households included in the baseline was eligible for follow-up in STAR. A key challenge was to identify who from the baseline survived and who perished in the disaster. We used three sources of information to determine whether a respondent had died: information from a member of the same household interviewed in the baseline; information from a neighboring household in the same enumeration area in the baseline; and information from expert community informants. For each respondent that we did not interview, we confirmed the mortality outcome in at least three waves and determined survival status for over 98 percent of the baseline sample [39]. Six percent of the baseline sample died in the tsunami [40]. Every baseline respondent who survived the tsunami was eligible to be interviewed in the follow-up surveys, including those who moved. Tracking respondents in any longitudinal survey that involves face-to-face interviews is a challenge.

Tracking respondents in the aftermath of a major disaster is extremely difficult but critically important since those who were displaced by the event are unlikely to be statistically exchangeable with those who stayed in the original location. Over 25 percent of the study sample was displaced or migrated away from their pre-tsunami residence. Nonetheless, over 96% of the tsunami survivors were interviewed in at least one of the post-tsunami follow-ups. This reflects the combination of three features of STAR. First, fieldwork is conducted by an extraordinarily dedicated team of Indonesians, all of whom are committed to collecting the highest quality data possible. Since the first post-tsunami follow-up, all fieldwork was under the direction of Dr. Cecep Sumantri of SurveyMETER, Indonesia. His outstanding leadership has been instrumental in the success of STAR. Second, our fieldworkers developed a rapport with the respondents, which is founded on mutual respect and trust, which has played a pivotal role in re-interviewing movers. Third, in collaboration with our Indonesian colleagues and drawing on our collective experience, we developed state-of-the-art information technology tools that facilitate tracking and assure field teams have the best information about the likely whereabouts of respondents at their fingertips at all times.

The pre-tsunami baseline provides detailed demographic and socio-economic information about household residents as well as information about respondents’ homes (ownership status and construction materials, for example). Post-tsunami follow-up surveys add extensive information on individual experiences of the tsunami (experiences as the waves came ashore, loss of family, loss of property), socio-economic status at each interview, physical and psychosocial health, and types and sources of assistance received. A specially designed community-level survey provided information about tsunami damage, humanitarian assistance, and reconstruction at the community level.

### 2.2. Measures

#### 2.2.1. Psychological Distress

We measured psychosocial distress with an index for post-traumatic reactivity constructed from the PTSD Checklist—Civilian Version (PCL), an instrument that has been validated in a range of settings [41,42]. During post-tsunami interviews, adult respondents were asked about the frequency and intensity of psychological reactions symptomatic of post-traumatic stress, such as repeated, disturbing memories, thoughts, dreams, or experiences of the tsunami and avoiding activities or situations because they reminded you of a stressful experience, where responses range from no experience (coded as 0) to frequent experience (coded as 3). Responses for each question were summed, resulting in a final PTSR scale that ranges from 0 up to 21 for those with the worst symptoms [43]. 

We also used the symptom reports to divide respondents into two groups as a function of the intensity of their distress, following criteria described by [41]. We define a binary outcome as one that takes the value of one if a respondent’s score is seven or higher and refer to that as elevated PTSR.

#### 2.2.2. Housing Assistance

We measured whether respondents received housing assistance and when they received it. At each post-tsunami interview, a respondent for the household was asked whether any member of the household had received a house, construction materials, or both, and if so, when. We created an individual-level measure of receipt of housing assistance based on whether a respondent was at the pre-tsunami baseline survey or a member of a household in which receipt of housing assistance was reported after the tsunami. This “intent to treat” measure does not restrict the indicator to include only respondents who were residing in an assistance house at the time of the survey.

We also constructed a measure of timing of receipt of housing assistance based on the household report of the month and year that benefits were first received. We cross-checked the household-level reports of receipt dates against the dates of housing reconstruction reported by community leaders to verify that household reports fall within the range of dates reported by community leaders. 

In about 38 percent of cases, household-level information on the receipt date of housing assistance was not available. Once housing assistance arrives in a community, people will receive houses around the same date. Accordingly, we developed an imputation algorithm that fills in missing values by randomly assigning a receipt date based on the dates provided by community residents who did report. The goal was to assign imputed dates so they match the distribution of the reported receipt dates.

#### 2.2.3. Covariates

Household-level covariates from the pre-tsunami baseline survey include whether, before the tsunami, respondents were living in a family-owned home; the roof and wall materials; expenditures (operationalized as the log of the monthly per capita level); and whether the residence was in an urban area. Individual-level variables include age, educational attainment, marital status, and whether the individual was directly exposed to the tsunami via seeing or hearing the waves, being caught up in the water, and/or watching family or friends struggle in the water. In addition, we used several other measures of exposure to the tsunami, including a variable that indicates whether the respondent’s baseline community experienced a high level of tsunami damage and whether the respondent’s home at baseline was damaged or destroyed at the time of the tsunami.

### 2.3. Study Design and Sample

All baseline respondents who survived the tsunami and were age 25 or older at the time of the pre-tsunami baseline were eligible for inclusion in this study. We exclude those respondents for whom we could not establish receipt of housing aid (2% of eligible adults), those for whom psychological health measures are incomplete (2.3%), and those for whom fewer than two post-tsunami measurements were completed (1.7%). Our analytical sample includes 12,447 adults interviewed in the baseline, with a total of 63,141 observations on PTSR and housing aid so that the average respondent was observed in 5 post-tsunami follow-ups. 

**Figure 2:**
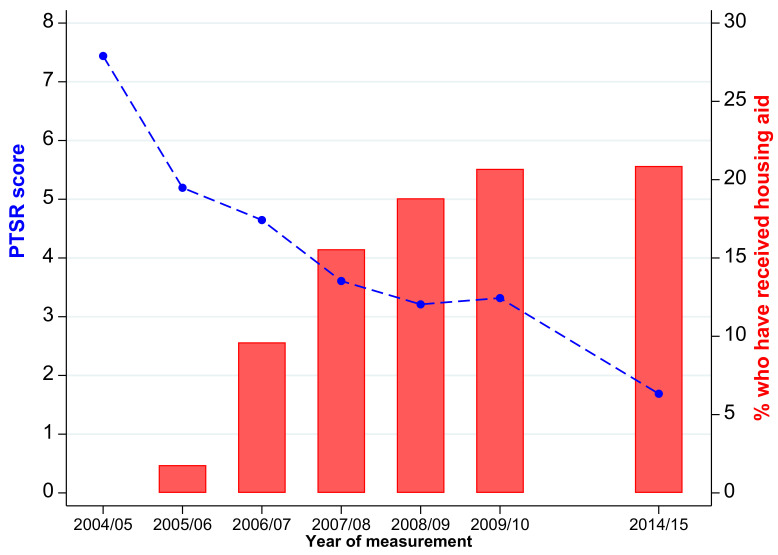
Evolution of PTSR and receipt of housing assistance in the ten years after the 2004 Indian Ocean Tsunami.

The evolution of PTSR and housing aid during the ten years after the tsunami are displayed in Figure 2. The red bars indicate the cumulative percentage of respondents who reported they had received housing aid by the time of each of the STAR follow-ups. As noted above, very little housing aid was distributed during the first 18 months following the tsunami. Distribution was ramped up during the following two years so that by the middle of 2008 (when the third STAR follow-up had been completed), aid had been distributed to three-quarters of the population who would ultimately receive housing aid. Aid distribution continued for another two years at a slower pace, and by the middle of 2010 (the fifth STAR follow-up), the provision of aid had largely ended. At that time, over 20% of the respondents reported having received some form of housing aid since the tsunami. 

The blue line in the figure traces the evolution of the average PTSR score (out of 21) of the study population measured at the time of each STAR follow-up. The population presented with high levels of PTSR in the first follow-up (an average score of 5.2), which declined in each of the following three years during the roll-out of housing aid. At that point, the decline halted, and PTSR rose slightly between the fourth and fifth follow-up. During the following five years, PTSR declined at an annualized rate that was slightly slower than the rate of decline immediately prior to the rise in PTSR. Ten years after the tsunami, the average PTSR score was 1.7, which is less than one-third of the level at the time of the first follow-up. 

The fact that the temporary halt in the secular decline of PTSR coincided with the end of the distribution of housing aid suggests the program may have contributed to improved psychological well-being; therefore, the two may be linked. However, the coincidence may also reflect other time-varying factors that drive both PTSR and housing aid, including, for example, the scaling down of the larger reconstruction program and the broader slow-down of the economy that occurred around the same time [44]. Our empirical methods are designed to provide evidence that distinguishes between these hypotheses. 

To lay a foundation that motivates those models, Table 1, Table 2 and Table 3 summarize the evolution of housing aid and PTSR disaggregated by measures of exposure to the tsunami. Two classes of exposure are key for this research. 

First, using high-resolution satellite imagery in conjunction with direct observation, we identified those STAR enumeration areas that were heavily damaged by the earthquake and tsunami. Specifically, using images from the Quickbird Satellite Sensor, we selected a 3 × 3 matrix of pixels around the center of each enumeration (about the size of three football fields) and compared an image taken two or three days before the tsunami with an image taken within three days after the tsunami. Based on whether pixels had turned to brown earth in combination with direct observations provided by our enumerators when they first visited each baseline enumeration area, communities were designated as heavily damaged. We identified 15% of the STAR enumeration areas as having been heavily damaged. 

Our second exposure indicator measured the effect of the tsunami and earthquake on the housing stock, and this measure is also specific to the respondent rather than measured at the community level. Specifically, in the first STAR follow-up, we asked a respondent in each household whether their house was damaged by the earthquake and/or tsunami and, if it was, whether it was completely destroyed. 

Table 1 presents the distribution of the sample by the extent to which their house was destroyed or damaged by the tsunami (in panel 1) and also by the extent of damage in the community in which they were a resident at the time of the tsunami (in panel 2). As shown in the first row of panel 1, houses were reported to have been destroyed for 39.7% of respondents, and house damaged was reported for another 23.2%. It is possible that respondents over-stated the extent of damage in the hope of getting housing aid. However, as shown in Figure 2, the first follow-up preceded the roll-out of the housing aid program, and it was not clear what aid would be forthcoming. (It was clear to respondents that our team would play no role in the allocation of aid.) Moreover, we repeated the same questions in the second follow-up, and test-retest reliability across the two waves was extremely high. 

Third, as shown in the second panel of Table 1, the probability a house being reported as destroyed was much higher in the communities that were heavily damaged: 79.1% of the houses were destroyed in those communities, whereas 32.5% of houses were destroyed in other communities. In fact, only 10.2% of houses in the heavily damaged communities were not damaged or destroyed.

The temporal path of the cumulative percentage of respondents who reported having received housing aid by the time of each STAR follow-up (displayed as red bars in Figure 2) is repeated in the first column of panel 1 of Table 2. The paths are displayed for the cumulative percentage of respondents who received housing aid among those whose houses were destroyed, damaged, or not damaged in columns 2, 3, and 4, respectively. 

Of those whose homes were destroyed in STAR, 42.2% reported receipt of housing aid. Of those whose houses were damaged, 11.1% received aid, and 4.8% received aid but did not describe their house as damaged. The roll-outs started earlier for those whose houses were destroyed: mean time to receipt of aid was just over 2 years after the tsunami. The roll-out was relatively rapid (90% of recipients had received aid within 33 months of the tsunami). The time since the tsunami that aid was received is displayed in panel 2 of the table. On average, those whose houses were destroyed received aid about a year before others. There is no difference in timing among those who reported their house was or was not damaged. 

The trajectory of the average PTSR score is displayed in panel 1 of Table 3. The percentage who reported elevated PTSR (score of at least 7 out of 20) is displayed in panel 2 of the table. Figure 2 documented the PTSR score declined for the entire sample except for the uptick 5 years after the tsunami. In the year after the tsunami, PTSR was highest among those whose homes were destroyed, and remained higher throughout the ten year period. While it was significantly higher relative to other respondents in the year after the tsunami, the gap shrunk and was not significant thereafter. Parallel patterns are reflected in the percentage of respondents who presented with elevated PTSR. The gap between those whose homes were destroyed and other respondents was large in the first few years after the tsunami. The uptick 5 years post-tsunami is striking, and since it is particularly large for those whose homes were not destroyed, there is no evidence of a gap in the longer term among those whose homes were destroyed or other respondents. 

Characteristics of the sample included in the analyses are reported in Table 4. All of the characteristics were measured in the pre-tsunami baseline. The average respondent was 40 years of age and had completed some junior high school (7.6 years of education). Half of the respondents were female. Four out of five were married before the tsunami, one in fourteen was a widow or widower at the time, and the rest had not yet married. 

Eighty percent of respondents lived in a home that they or another household member owned. In order to adjust for pre-tsunami resources at the household level, we used monthly per capita expenditure (PCE), which is generally regarded as a better indicator of resource availability than, for example, income, which is relatively volatile. The average respondent lived in a household with PCE of IDR 370,000 (which is about USD 30). Because PCE is right-skewed, we included the logarithm of PCE in the multivariable models.

### 2.4. Empirical Specifications

Our empirical methods were designed to, firstly, identify those who received housing and, secondly, document the relationship between receipt of housing assistance and levels of post-traumatic stress. Establishing a causal relationship between receipt of housing aid and stress is complicated if aid is not randomly allocated: we provided evidence that aid and the timing of aid were targeted to specific individuals and communities. Therefore, we leveraged the combination of the timing of aid and the longitudinal data on PTSR to identify the effect of post-disaster recovery assistance on psychological well-being.

We began with an investigation of the characteristics that predicted receipt of housing aid and its timing, focusing on the role of tsunami exposure while taking into account individual and household characteristics along with pre-tsunami residential location. We estimated individual-level multivariable models of the probability a respondent had received housing aid by the time of the 10-year STAR follow-up and, among those who received aid, the number of months that elapsed between the tsunami and aid receipt, $A_{i}$.

(1) $$A_{i}=E_{i}\alpha_{1}+X_{c}\alpha_{2}+X_{i}\alpha_{3}+\mu_{c}+\upsilon_{ic}$$

The models include both indicators of tsunami exposure; $E_{i}$: whether the community in which the respondent was living sustained heavy damage and whether the respondent’s house was destroyed or damaged. All models also take into account the location of the house by including distance to the capital city, Banda Aceh; whether the area was urban; distance to the coast and elevation, $X_{c}$*;* as well as *kecamatan* (sub-district) fixed effects, $\mu_{c}$. These controls contribute to isolating the effect of the exposure indicators and sweep out the role of other community-level factors that are correlated with damage, access to resources, and central decision making. In addition, the models include individual and household socio-economic and demographic characteristics measured in the pre-tsunami baseline, $X_{i}$, to provide evidence about the distribution of aid within the population. Unobserved heterogeneity is reflected in $\upsilon_{ic}$ and permits both individual-level heterogeneity and common, unobserved factors at the community level.

We next turned to the relationship between housing aid and PTSR. Establishing a causal link is complicated because aid is not likely to be allocated randomly, in which case an estimate based on a comparison of PTSR of those who received aid with PTSR of those who did not receive aid will likely yield a biased estimate of the effect of post-disaster recovery assistance on psychological well-being. To address this concern, we developed a difference-in-difference estimation procedure designed to identify the causal effect of housing aid on PTSR. Intuitively, the trajectory of PTSR after receipt of aid is compared with the trajectory of PTSR before receipt of aid for the same individual after differencing out all individual-specific fixed characteristics that affect both PTSR and receipt of aid.

Specifically, we leveraged the fact that the timing of aid, $A_{it}$, was driven by a plethora of administrative and logistical factors in combination with the longitudinal dimension of STAR, in which we measured PTSR, $P_{it}$, multiple times for the same individual both before and after the receipt of aid:

(2) $$P_{it}=A_{it}\beta_{1}+X_{ct}\beta_{2}+X_{it}\beta_{3}+\eta_{i}+\mu_{ct}+\varepsilon_{ict}$$

The model includes an individual-specific fixed effect, $\eta_{i}$, which absorbs all observed and unobserved factors at the individual level that affect PTSR and the receipt of aid and are fixed during the study period (i.e., between the STAR follow-up in the year after the tsunami and the 10-year follow up). These include, inter alia, the individual’s experience of the tsunami, whether the individual was eligible for aid because their house was destroyed or damaged, whether the individual was more likely to receive aid because of connections or resources, along with background and genetic factors that are related to psychological well-being.

A survey-wave fixed effect would absorb secular changes in PTSR and aid receipt that are common across the study areas. However, as shown in Table 1, time trends differed across space, and so, model (2) also includes *kecamatan*-specific survey-wave fixed effects, $\mu_{ct}$. The combination of individual fixed effects and *kecamatan*-wave fixed effects means that comparisons are drawn between PTSR of an individual after receipt of aid with the same individual’s PTSR prior to receipt of aid, and this difference was compared with the difference in PTSR between the same two survey waves for someone who was living in the same *kecamatan* at the time of the tsunami and did not receive aid. The difference in differences estimates, ${\hat{\beta }}_{1}$, are designed to measure the causal effect of receipt of aid on PTSR.

In addition, to assure that these difference-in-difference estimates were not contaminated by time-varying characteristics within each *kecamatan*, the models include indicators of reconstruction during the study period, $X_{ct}$*,* measured at the enumeration area level in every survey wave. These include whether, at the time of the survey wave, the community had received block grants, public works programs, reconstruction of roads and bridges, and reconstruction of health facilities. In addition, the models include two time-varying individual-specific characteristics that are potentially important for PTSR: they are the respondent’s marital status, and level of household resources, *ln*(PCE), $X_{it}$.

Unobserved heterogeneity was captured by $\varepsilon_{ict}$. Under the assumption that this unobserved heterogeneity in the model is not correlated with aid receipt and its timing, after taking into account the two levels of fixed effects and time-varying controls, the estimates in (2) can be assigned a causal interpretation.

Using all six post-tsunami waves of STAR collected between 2005 and 2010, model (2) was estimated with two indicators of psychological well-being, $P_{it}$, measured in each wave: the PTSR score and an indicator for whether the score was 7 or higher. Receipt of aid was measured in three ways to fully leverage the specific timing of the aid. In the first method, *A_it_* is an indicator variable taking the value one if the respondent had received aid by the time of the survey interview and zero otherwise. This method amounts to comparing average PTSR after aid receipt with the average before aid receipt. To more fully leverage the precise timing of receipt, the second method measured the time (in years) since the aid was received. It was not obvious that the impact of aid should vary linearly with time, and so, in the third method, indicator variables were included for receipt of aid at least two years before the survey interview, 6–24 months before the interview, and within 6 months of the interview. To allow for the possibility that people may have known they would receive aid in the near future, the models also include an indicator for receipt of aid in the 6 months following the interview. If there is no prior knowledge of aid receipt, or if expectations about future aid are noisy, then this indicator of future receipt provides a placebo test for the model specification. In this third specification, the estimated effects are relative to the absence of aid within 6 months of the survey interview. 

The models were estimated by the method of ordinary least squares (OLS). Logistic regression estimates of the probability of aid receipt and the probability that PTSR equals or exceeds 7 yield the same conclusions as the OLS estimates. Estimates of standard errors take into account heteroskedasticity of arbitrary forms and clustering of sampled respondents at the level of the baseline community [45].

## 3. Results

### 3.1. Predicting Receipt of Housing Assistance

Table 5 presents results for estimates of model (1), which predict whether a respondent ever received aid, and for those who received any aid, the time (in months) since the tsunami that aid was received are in columns 1 and 2, respectively. Estimates in the first column are multiplied by 100 so that they can be interpreted in terms of percentages. Estimated standard errors are reported in parentheses below the coefficient estimates.

As shown in panel A of the table, controlling individual, household, and community characteristics, respondents who were living in an area that was subsequently heavily damaged by the tsunami were 18% more likely to receive aid than those who were living in other areas, and the aid was received 7.5 months earlier in the heavily damaged communities. If the respondents’ house was destroyed, they were 19% more likely to receive aid, and aid was distributed 8 months earlier. Since these effects are additive, a respondent whose house was destroyed and was living in a community that was heavily damaged was nearly 40% more likely to receive aid than someone living elsewhere whose house was not damaged. If the respondent’s house was damaged but not destroyed, they were 5% more likely to receive aid than someone whose house was not damaged, and there was no difference in timing of aid. 

There is no evidence that urban areas were privileged in receipt of aid or its timing, nor were home-owners or those with houses that were built of better materials (panel B). There is evidence, however, that respondents with more household resources, as measured before the tsunami, were less likely to receive aid: those who were in the top quartile of PCE were 6.7% less likely than those in the bottom quartile, and the difference was significant for all whose PCE was above median (panel C). In practice, housing aid was targeted towards those who were the poorest before the tsunami. Aid was also targeted towards younger tsunami survivors: those who were age 25–34 years at the time of the tsunami were about 3% more likely to receive aid. 

While resources and age are significant predictors of aid receipt, their effect sizes are modest relative to the differences due to housing damage and residential location. None of the other socio-demographic characteristics was a significant predictor of receipt of aid or its timing. (There is a suggestion that those who were widowed pre-tsunami were slightly more likely to receive aid, but the estimate is small and only significant at a 10% size of test.)

### 3.2. Impacts of Housing Assistance on Post-Traumatic Stress Reactivity

Table 6 presents results for estimates of model (2) for the PTSR score (in column 1 of each panel) and an indicator for those who present with elevated PTSR (≥7) (column 2). The estimates for elevated PTSR are multiplied by 100 and are interpreted as percentage effects.

All respondents are included in the models in the first two columns (section I) of the table. Since effects of aid are likely to be greatest for those whose homes were destroyed, in section II, respondents are stratified by whether or not their house was destroyed.

Panel A displays results for models that include an indicator defined as one if the respondent received aid at the time of the STAR interview when PTSR was measured and zero otherwise. Since the models include individual fixed effects, respondents who did not receive aid did not contribute to the estimation. The effects of aid were identified by comparing (average) PTSR of an individual in the interviews after aid was received with their (average) PTSR in the interviews before aid was received. Aid receipt is associated with a 1.2 percentage point reduction in the probability a respondent presents with elevated PTSR (column 2). This effect is both statistically significant and large in magnitude (relative to the mean of 2.6 two years post-tsunami). PTSR average scores (column 1) are also lower after receipt of aid, but the effect is small in magnitude and not statistically significant. As shown in the second column of section II.1, the effect on elevated PTSR is concentrated among those whose homes were destroyed, and the effect size is larger (1.7 percentage points), albeit off a larger base.

These estimates compare PTSR levels of the same person before and after aid receipt, the timing of which varied substantially across the study population. Further, the change between waves for an individual who received aid was compared with the change between the same waves for another individual who lived in the same *kecamatan* at the time of the tsunami but did not receive aid between the waves. These difference-in-difference estimates also take into account time-varying indicators of community-level receipt of other types of assistance and time-varying, individual-specific characteristics.

To more fully leverage the specific timing of aid receipt, in panel B.1 of the table, we show a dose–response relationship that examines how the trajectory of PTSR varies with time since the receipt of aid. Both PTSR scores and the probability that PTSR is elevated declined with time since aid was received. In both cases, the estimated effects are statistically significant. Again, the effects were larger for those whose homes were destroyed and not statistically significant for others. For example, for each year since aid was received, the probability that a respondent whose home was destroyed presented with elevated PTSR was 0.34 percentage points lower. 

Under the very strong assumption that receipt of aid had no effect on psychological well-being of those whose homes were not destroyed, then the estimate for that group (0.05 in II.2 column 2) can be interpreted as a time effect. It is both very small in magnitude and not significant: it provides an upper-bound estimate of the potential contamination from unobserved heterogeneity that is correlated with the model covariates. We conclude that contamination from time effects is likely to be modest in the model, providing additional support for giving the estimated effects of aid on PTSR a causal interpretation.

It is not clear the effects of time since receipt of aid should be linear. Panel B.2 presents estimates with indicator variables for time periods so that the effects can vary with time. There is no evidence that aid has any impact on psychological well-being in the first six months after receipt or that the expectation of aid affects psychological well-being. However, in models that include all respondents by 6 months, there is a substantial improvement in both PTSR and the probability of presenting with elevated PTSR, and these effects are large in magnitude and statistically significant two years post-receipt. The magnitude of the effects on PTSR scores of those whose homes were destroyed are slightly larger but less well-determined, and they are not statistically significant. The effects on presenting with elevated PTSR are both larger in magnitude and statistically significant (for receipt that was 6–24 months and at least 2 years before the STAR interview). The effects on those whose homes were not destroyed are, again, much smaller and not statistically significant. This provides further assurance that contamination from time effects is unlikely to be important. 

## 4. Conclusions

Although billions of dollars are spent annually on assistance and reconstruction after disasters strike, much less attention has focused on whether provision of stable housing in the aftermath of a disaster ameliorates psychological distress and improves longer-term recovery outcomes. Our paper makes an important contribution to the literature by evaluating the potential of post-disaster assistance programs to affect population well-being. Focusing on the role of housing assistance in Aceh, Indonesia, in the aftermath of the 2004 Indian Ocean tsunami, the research has provided several important conclusions. 

First, the housing reconstruction program’s benefits were targeted to communities in which damage was substantial and to individuals who experienced housing damage and destruction. On average, those with fewer economic resources before the tsunami were more likely to receive housing assistance in its aftermath and to receive it more quickly. These findings are important given frequently cited concerns and in some cases empirical evidence that assistance money fails, for various reasons, to find its way to the areas of greatest damage and to the neediest victims [46,47]. Though provision of post-disaster housing in ways that are efficient, cost-effective, and well-targeted is complicated, our results show that housing programs can succeed at reaching their targets. 

We then turned to whether policy responses in the form of provision of housing can improve psychological outcomes. We showed that receipt of housing is associated with a significant reduction in symptoms of post-traumatic stress and in scoring above the cutoff for high PTSR. These results are from models that include individual fixed effects and so are robust to unobserved factors that predict both receipt of housing and psychosocial health. The models include place-survey wave-fixed effects and time-varying community covariates and so are robust to declines in PTSR as time since the tsunami increases. By exploiting our data on timing of receipt of housing assistance, we also showed that positive impacts grow over time, are strongest two or more years after receipt of housing, and are concentrated among those whose homes were destroyed in the event. 

Our research is one of few analyses designed to establish a causal link between reconstruction and post-disaster individual outcomes. The findings have important public health implications in the context of helping populations recover from disasters. They show that the rapid deployment of resources toward provision of permanent housing can substantially reduce symptoms of post-traumatic stress. The impact of housing assistance is strongest for people who lost their home in the tsunami, further underscoring the importance of helping people get back into stable housing when disaster strikes. Our analysis underscores another important methodological point. We documented that benefits are greatest for those who received housing assistance two or more years in the past, followed by those who received housing aid between six months and two years ago. Studies that focus only on short-term effects will understate program benefits if the benefits cumulate over time, as they do in this case. 

Finally, results from this research extend beyond reconstruction post-disaster. More broadly, our results show that policies aimed at securing adequate housing and protecting economic wealth can have long-lasting significant effects, minimizing the psychosocial effects of negative shocks.

## Figures and Tables

**Table 1:** Tsunami exposure and housing damage.

	All	Tsunami Resulted in House Being		
	Respondents	Destroyed	Damaged	Not Damaged
	(1)	(2)	(3)	(4)
1. All respondents				
Percentage	100.0	39.7	23.2	37.1
Number	12,447	4944	2885	4618
2. Respondents by extent of damage to community of residence at time of tsunami				
2.1 Community was heavily damaged				
Percentage	15.6	79.1	10.7	10.2
Number	1936	1532	207	197
2.2 Other communities				
Percentage	84.4	32.5	25.5	42.1
Number	10,511	3412	2678	4421

**Table 2:** Receipt of housing aid and tsunami-related housing damage.

	All	Tsunami Resulted in House Being		
	Respondents	Destroyed	Damaged	Not Damaged
	(1)	(2)	(3)	(4)
1. % of respondents who received housing aid by year of survey follow-up				
Years since tsunami:				
1	1.8	3.5	0.6	0.5
2	9.6	20.2	3.1	1.9
3	15.6	33.1	6.3	2.6
4	18.8	38.9	8.4	3.6
5	20.7	41.9	11.0	4.3
10	20.9	42.2	11.1	4.8
2. Time from tsunami (in months) at receipt of housing aid				
Mean	28.1	25.9	37.3	35.4
Std dev	(16.9)	(13.5)	(23.2)	(25.7)
10%ile	12	11	14	8
90%ile	36	33	49	47
No of respondents	12,447	4944	2885	4618

**Table 3:** PTSR by tsunami-related housing damage and years since tsunami.

	All	Tsunami Resulted in House Being		
	Respondents	Destroyed	Damaged	Not Damaged
	(1)	(2)	(3)	(4)
1. Mean and (standard deviation) of PTSR score (max = 21)				
Years since tsunami:				
1	5.2	5.9	4.9	4.6
	(3.6)	(3.8)	(3.5)	(3.2)
2	4.6	5.1	4.7	4.1
	(3.9)	(3.9)	(3.9)	(3.7)
3	3.6	4.0	3.5	3.2
	(3.6)	(3.7)	(3.6)	(3.4)
4	3.2	3.4	3.1	3.0
	(3.3)	(3.4)	(3.2)	(3.2)
5	3.3	3.6	3.3	3.1
	(3.4)	(3.6)	(3.4)	(3.3)
10	1.7	1.8	1.6	1.6
	(2.9)	(3.0)	(2.8)	(2.8)
2. % of respondents who present with PTSR score 7 or higher				
Years since tsunami:				
1	2.6	4.1	1.8	1.2
2	2.6	3.4	2.9	1.5
3	1.5	2.0	1.2	1.2
4	0.9	1.2	0.5	0.7
5	1.5	1.5	1.6	1.5
10	0.8	0.9	0.7	0.8
No of respondents	12,447	4944	2885	4618

**Table 4:** Characteristics of respondents measured at pre-tsunami baseline.

	Mean	Std. Dev.
1. Individual characteristics		
Age (years)	40.8	(11.9)
Years completed education	7.6	(4.5)
% who are female	50.9	
% who were head of household	47.3	
% who were married	79.9	
% who were widowed	7.4	
2. Household characteristics		
% owned home	81.3	
Household monthly PCE (IDR 000)	370.0	(312.8)
Ln (PCE)	12.6	(0.6)
Sample size	12,447	

**Table 5:** Predictors of receipt of housing aid and timing of receipt of aid.

	Ever Received Aid (%)	No of Months after Tsunami Received Aid
	(1)	(2)
A. Tsunami exposure		
Community level damage		
(1) If community heavily damaged	18.22 **	−7.46 **
	(4.50)	(2.47)
Household level damage		
(1) If house destroyed in tsunami	19.37 **	−8.04 **
	(1.78)	(2.44)
(1) If house damaged in tsunami	5.30 **	−0.91
but not destroyed	(1.07)	(2.68)
B. Pre-tsunami housing characteristics		
(1) If owned house pre-tsunami	−0.02	−1.06
	(1.24)	(1.37)
(1) If pre-tsunami house roof tiled	−1.54	−0.95
	(2.53)	(2.09)
(1) If pre-tsunami house walls brick	−0.21	−0.58
	(1.11)	(0.94)
(1) If area was urban pre-tsunami	−1.82	0.80
	(2.52)	(1.70)
C. Household ln(PCE) at pre-tsunami baseline		
(1) If 26–50 percentile	−2.12	0.60
	(1.53)	(1.25)
(1) If 51–75 percentile	−4.53 **	1.04
	(1.62)	(1.34)
(1) If top quartile	−6.71 **	2.50
	(1.82)	(1.61)
D. Individual characteristics at pre-tsunami baseline		
*Age*		
(1) If age 35–44 y	−3.15 **	0.46
	(0.88)	(0.84)
(1) If age 45–99 y	−3.46 **	0.96
	(1.11)	(1.00)
*Education*		
(1) If completed 1–6 y education	−0.80	1.38
(Primary school)	(1.39)	(1.23)
(1) If completed 7–9 y education	−2.68	1.48
(Junior high school)	(1.56)	(1.43)
(1) If completed at least 10 y education	−3.27	0.45
	(1.70)	(1.51)
*Demographics*		
(1) If female	−1.33	0.43
	(0.90)	(0.97)
(1) If head of pre-tsunami HH	−0.61	0.55
	(0.98)	(1.00)
(1) If married pre-tsunami	−1.20	−2.06
	(1.14)	(1.17)
(1) If widowed pre-tsunami	2.75	−4.09 *
	(1.78)	(1.81)
R^2^	0.42	0.37
N	12,447	2629

Note: ** *p* < 0.01, * *p* < 0.05. Models estimated by OLS. All models also control distance to the coast, elevation distance to Banda Aceh, distance to the earthquake epicenter, *kecamatan* (subdistrict)-fixed effects, and household composition. Excluded categories for indicator variables are bottom quartile of PCE, age 25–34 years, and no education. Underscored and italicized headings indicate groups of covariates. Robust standard errors in parentheses take into account clustering at pre-tsunami community level and heteroscedasticity of arbitrary form.

**Table 6:** Relationship between PTSR and housing assistance receipt as well as timing of assistance.

	I. All Respondents		II. Was Home Destroyed in the Tsunami?			
			II.1. Yes		II.2. No	
	PTSR (score)	(1) If PTSR ≥ 7	PTSR (score)	(1) If PTSR ≥ 7	PTSR (score)	(1) If PTSR ≥ 7
	(1)	(2)	(1)	(2)	(1)	(2)
A. Whether received housing aid						
(1) If received aid at time of interview	−0.21	−1.21 *	−0.21	−1.66 *	−0.15	−0.52
	(0.12)	(0.51)	(0.15)	(0.71)	(0.22)	(0.92)
B. Timing since receipt of housing aid						
B.1 Time (measured in years)						
Time since receipt of aid	−0.06 **	−0.23 **	−0.08 **	−0.34 **	−0.04	−0.05
	(0.02)	(0.08)	(0.03)	(0.12)	(0.04)	(0.15)
B.2 Indicator variables for time since receipt of aid						
(1) If received aid at least 2 years ago	−0.31 *	−2.01 **	−0.37	−2.84 **	−0.18	−0.82
	(0.14)	(0.62)	(0.20)	(0.87)	(0.25)	(1.03)
(1) If received aid 6–24 months ago	−0.23	−1.29 *	−0.29	−1.76 *	−0.17	−0.60
	(0.14)	(0.61)	(0.17)	(0.79)	(0.27)	(1.06)
(1) If received aid within 6 months	0.11	−0.18	0.04	−0.43	0.15	−0.64
	(0.14)	(0.64)	(0.16)	(0.84)	(0.31)	(1.17)
(1) If will receive aid in next 6 months	0.04	−0.45	−0.05	−0.86	0.13	0.63
	(0.18)	(0.94)	(0.21)	(1.21)	(0.39)	(1.71)
Number of respondent-wave observations	63,141	63,141	25,315	25,315	37,826	37,826

Note: ** *p* < 0.01, * *p* < 0.05. Each horizontal panel in each column is a separate model estimated using all observations for each panel respondent assessed in post-tsunami waves. All models include individual fixed effects, *kecamatan* of residence at time to tsunami, fixed effects interacted with survey wave, time-varying community characteristics (block grants, public works programs, rebuilding transport infrastructure, and rebuilding of health facilities), and controls for marital status and ln (household per capita expenditure) in each wave. Robust standard errors clustered at pre-tsunami community level.

## Data Availability

Data from the Study of the Tsunami Aftermath and Recovery (STAR) are available at stardata.org (accessed on 30 April 2022).
